# Ionic Liquids and Ohmic Heating in Combination for Pd-Catalyzed Cross-Coupling Reactions: Sustainable Synthesis of Flavonoids

**DOI:** 10.3390/molecules25071564

**Published:** 2020-03-29

**Authors:** Vera L. M. Silva, Raquel G. Soengas, Artur M. S. Silva

**Affiliations:** 1 LAQV-REQUIMTE, Department of Chemistry, University of Aveiro, 3810-193 Aveiro, Portugal; verasilva@ua.pt; 2 Department of Organic and Inorganic Chemistry, University of Oviedo, c/Julián Clavería 6, 33006 Oviedo, Spain; rsoengas@uniovi.es

**Keywords:** ionic liquids, ohmic heating, cross-coupling, flavonoids

## Abstract

In order to meet the increasing demand for environmentally benign chemical processes, we developed a Suzuki–Miyaura reaction protocol based on the combination of ohmic heating (ΩH) and supported ionic liquid phase catalysis (SILPC) in aqueous media. This methodology was applied to the synthesis of a series of flavonoid derivatives, including isoflavones, styrylisoflavones, and diarylalkenylisoflavones.

## 1. Introduction

Pd-catalyzed cross-coupling reactions exemplify one of the most powerful and popular methods for the formation of carbon–carbon bonds [1]. The importance of these reactions has driven chemists to explore alternative procedures. Out of these, some have been truly game-changing, dramatically accelerating development in the field. One such report appeared in 2000 and described a Suzuki coupling using the ionic liquid (IL) [bmim][BF_4_] as reaction media. Inspired by this pioneering work, several research groups became interested in metal-containing ionic liquids for cross-coupling reactions. That led to a crucial finding—metal-containing ionic liquids efficiently promote ligand-free cross-coupling reactions, in which ILs can serve as both immobilization solvent and ligand to the catalysts [2]. The metal-containing IL is highly recyclable and can be used in several cycles without loss of activity.

For the use in industry, solid catalysis with ILs would be the ideal choice for cross-coupling reactions, avoiding the chance of product contamination by the metal. Thus, the concept of supported IL phase catalysis (SILPC) for transition metal-catalyzed cross-coupling reactions has gained much recent attention. Covalently grafting ILs onto the surface of solid materials not only heterogenizes the expensive IL, but also offers a lot of opportunities to investigate immobilizations of homogeneous metal complexes or metal nanoparticles [3]. In addition to acting as co-catalysts able to modify the activity of palladium species present in catalytic systems, ILs are known stabilizing agents for Pd(0) nanoparticles, observed in most catalytic systems containing palladium precursors without phosphorus ligands [4]. The solid-supported IL would immobilize and stabilize the formed Pd(0) nanoparticles, which can be then recovered from the catalytic mixture and reused with almost unchanged activity. A further advantage of basing Pd-catalyzed cross-coupling reactions in SILPC is that such reactions are usually performed in water and are significantly enhanced by microwave (MW) heating. However, MW heating has some important limitations that remain unsolved. One important drawback is related to the low efficiency of the magnetron in converting electrical energy into microwave energy (about 50–60%) [5]. Thus, in the case of reflux using an open vessel system on a small scale, microwave radiation consumes significantly more energy than conventional heating (mantle or oil bath). In addition, scaling-up reactions under MW heating is problematic due to the limited penetration depth of microwave radiation in absorbing media. This fact, together with safety concerns, makes MW heating not suitable for its application to industrial scale [6].

**Figure 1:**
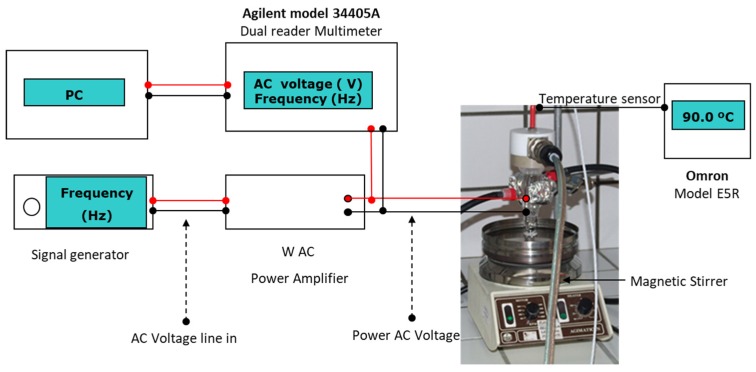
Schematic representation of the ohmic heating reactor.

In order to overcome these limitations, we have developed in our group an alternative based on the use of ohmic heating (ΩH) for chemical reactions [7,8]. In this thermal process the reaction mixture, which serves as an electrical resistor, is heated by passing electricity through it (electrodes are in contact with the conductive reaction medium). Thermal energy is generated by the motion of the charged species in solution as a result of the high frequency (25 kHz) AC electric current. The thermal-energy transfer occurs mostly between the electrode plates cross-session region and surroundings. Electrical energy is dissipated into heat with high efficiency, providing a high-speed heating rate, rapid, and uniform heating (temperature homogeneity), as well as an enhancement of the charged-species dynamics, which can conduct to a distinct reactivity path. ΩH leads to the same effects as MW activation on the reaction, reducing the reaction time and also activating metal surface and facilitating single electron transfer (SET) reactions [9]. Our ohmic-reactor operates at atmospheric pressure, under open-vessel conditions, and typically at the boiling temperature of the solvent (Figure 1) [10].

Given that ΩH is ideally suitable for synthesis in aqueous media, in which the reaction mixture is electrically conductive, water in combination with SILPC is a propitious solvent for this process. On the other hand, the combination of ΩH and water as solvent provides great opportunities for sustainable chemistry. Water is non-toxic, non-flammable, and is easily available at low cost. In addition, water has a high thermal capacity, making the exothermic processes safer and more selective, especially when they are performed on a large scale. Moreover, ohmic heating is already used in industry, especially for food processing, with several major equipment manufacturers offering commercial ohmic heaters, serving a growing market of food manufacturing companies, so the combination of ΩH and SILPC in aqueous media may be of interest not only from the perspective of green chemistry but also of industrial scalability.

In order to demonstrate the usefulness of this strategy, we envisioned its application to the synthesis of the biologically relevant class of compounds such as flavonoids.

Flavonoids are a family of natural products present as secondary metabolites in plants, which display remarkable biological activity including antimalarial, anticancer, antioxidant, and anti-inflammatory activities [11,12,13,14,15,16,17,18,19,20]. Due to their biological profile, numerous approaches have been reported for the synthesis of flavonoid derivatives. Among them, palladium-catalyzed cross-coupling reactions are gaining much recent interest [21,22,23,24,25,26,27], the Suzuki–Miyaura coupling reaction being the most frequently used methodology [28]. In this regard, ΩH-enhanced cross-coupling reactions, and especially Suzuki–Miyaura reactions, have proven particularly useful for the synthesis of pharmacologically relevant scaffolds [29,30].

Herein we describe the combination of ΩH and SILPC for the Suzuki cross-coupling reaction and its application to the synthesis and functionalization of flavonoids.

## 2. Results and Discussion

We started our studies with the immobilization of the Pd catalyst. For this purpose, we used the immobilization procedure described by Hagiwara et al., in which Pd(OAc)_2_ was supported on amorphous silica with the aid of an imidazolium ionic liquid ([bmim]PF_6_) [31,32]. The procedure of immobilization is quite simple; a suspension of spherical amorphous silica in a solution of Pd(OAc)_2_ in [bmim]PF_6_ and CH_2_Cl_2_ was evaporated to dryness and washed with diethyl ether, to afford a powdery and free-flowing immobilized catalyst (Pd@SILP). Structural studies suggest that the catalyst consists of a solution of Pd(OAc)_2_ in [bmim]PF_6_ physisorbed in the SiO_2_ pores [31].

Initially, the Suzuki–Miyaura reaction under the supported Pd catalyst was assessed with the coupling of 3-bromochromone **1a** and phenylboronic acid **2a** under various conditions, and the results are compiled in Table 1. Water was identified as the optimal solvent; combined with TBAB as PTC (phase transfer catalyst) furnished the desired isoflavone **3a** in 89% yield via conventional heating at 100 °C for 12 h (Table 1, entry 6). With the optimized conditions in hand, we investigated the effect of the use of ohmic heating on the yield and the reaction rate. Thus, when 0.5 mmol of **1a** was allowed to react with 0.6 mmol of phenylboronic acid **2a** in the presence of 0.5 mmol of Na_2_CO_3_ as a base, 0.1 mol% of TBAB (tetrabutylammonium bromide) as PTC (phase transfer catalyst) and 1 mol% of Pd-supported catalyst in water at 100 °C under ohmic heating, the reaction proceeded in 1 h and afforded isoflavone **3a** in an excellent 93% yield (Table 1, entry 7). Decreasing the catalyst loading to 0.1 mol% did not significantly affect the yield (Table 1, entry 8). The catalyst loading can be further decreased to 0.05 mol%; under such conditions, isoflavone **3a** was formed in a 90% yield (Table 1, entry 9).

The work-up procedure is very simple: the reaction mixture was filtered, washing thoroughly with water. After extraction with diethyl ether, ^1^H NMR of the crude reaction product indicated that **1a** was fully transformed to the isoflavone **3a**.

The application of the optimized protocol to a range of flavonoid bromides is described in Table 2. In a typical procedure, a mixture of flavonoid bromide **1** (0.5 mmol) and aryl boronic acid **2** (0.6 mmol) in water was heated under ohmic heating at 100 °C in the presence of TBAB (0.1 mol%), Na_2_CO_3_ (0.5 mmol) and Pd@SILPC (0.05 mol%) for 1 h.

Coupling of **1** proceeded smoothly regardless of the electronic nature of the substituents. Thus, the coupling reaction of **1a** and both electron-deficient and electron-rich arylboronic acids afforded in all cases the corresponding isoflavones **3a**–**c** in high yield (Table 2, entries 1–3). Bromovinyl chromones **1b**,**c** also underwent the coupling reaction with boronic acids **2a**,**b** affording 3-styrylchromones **3d**–**f** in good yields (Table 2, entries 4–6). Interestingly, the coupling of 2.0 equiv. of arylboronic acids **2b**,**c** with *gem*-dibromovinyl bromoflavones **1d**–**g** afforded the corresponding bisarylvinyl chromones **3g**–**j** in good yields. To the best of our knowledge, the biological profile of this family of chromone derivatives remains unexplored, which is surely related to the lack of an efficient procedure for the synthesis of these flavonoid derivatives. In fact, there is just one example in the literature in which bisarylvinyl chromones are obtained in low yields by the reaction of formyl chromone and ketene. [33]

The lifetime of the catalyst and its level of reusability are very important factors for practical applications. In order to evaluate this issue, we envisioned a series of experiments using the recycled catalyst. Thus, we performed the reaction of 3-bromochromone **1a** with phenylboronic acid **2a** on a 5 mmol scale. After the completion of the first reaction to afford the corresponding isoflavone **3a** in 93% yield, the catalyst was recovered by filtration, washing the reaction vessel and electrodes thoroughly with distilled water and *n*-pentane. After drying the recovered catalyst in vacuum at 40 °C for 5 h, a new reaction was performed with fresh solvent and reactants under the same conditions. Gratifyingly, the supported Pd@SILP system could be reused three times with no considerable loss of activity, affording isoflavone **3a** with an average chemical yield of 88.3%. In addition, isoflavone **3a** was analyzed through ICP-MS, showing negligible Pd contamination (3.48 ppb).

## 3. Materials and Methods

NMR spectra were recorded on a 300 MHz NMR spectrometer [300.13 MHz (1H), 75.47 MHz (13C)] with TMS as an internal reference and with CDCl_3_ as a solvent. Chemical shifts (*δ*) are quoted in ppm relative to TMS. Coupling constants (*J*) are quoted in Hz. Mass spectra analysis (ESI-MS) and high-resolution mass spectra analysis (ESI-HRMS, 70 eV) were carried out on an electrospray ionization mass spectrometer with a micro-TOF (time-of-flight) analyzer. For experiments carried out under ohmic heating, the 10 mL reactor was filled with the reaction mixture closed, and the mixture was heated to 100 °C. For 4 mL of reaction mixture the length of electrodes immersed in the reaction medium was 9 mm; the distance between the electrodes was 10 mm. Medium magnetic stirring speed (740 rpm) was used in all the experiments carried out in the ohmic heating reactor. Temperature measurement was done using a type J glass-sheathed thermocouple inside the reactor. For the experiments carried out in conventional heating (oil bath), medium magnetic stirring speed (740 rpm) was used.

### 3.1. Representative Procedure for Catalyst Preparation

To a stirred solution of Pd(OAc)_2_ (12 mg) in [bmim]PF_6_ (0.1 g) and CH_2_Cl_2_ (10 mL) was added silica powder (1 g, spherical for flash column chromatography, diameter; 40–50 mm, pore size; 5–7 nm, pore volume; 0.80–1.00 mL/g, surface area; 600–700 m^2^/g). After being stirred for 90 min at room temperature, CH_2_Cl_2_ was evaporated to dryness to give a light brown dry powder, which was rinsed with diethyl ether and dried in vacuo to afford the Pd supported catalyst (~1% wt based on weight gain).

### 3.2. General Procedure for the Suzuki–Miyaura Reaction.

A mixture of the corresponding bromochromone **1** (0.50 mmol), the corresponding boronic acid **2** (0.60 mmol), Na_2_CO_3_ (0.50 mmol) and Pd@SILP (0.05% mmol of Pd) in H_2_O was stirred under ohmic heating at 100 °C for 1 h. The catalyst was filtered-off and the filtrate was extracted with Et_2_O (3 × 10 mL). The combined extracts were dried over Na_2_SO_4_ and evaporated under reduced pressure affording products **3**. The physical data of known isoflavones **3a**–**f** were comparable to those of the literature [34,35,36,37,38]. The physical data of the new isoflavones **3g**–**j** are shown below.

**3-[2,2-Bis(3,4-dimethoxyphenyl)vinyl]-4*H*-chromen-4-one (3g):**^1^H NMR (300 MHz, CDCl_3_) δ 8.27 (ddd, *J* = 8.0, 1.7, 0.5 Hz, 1H), 7.63 (ddd, *J* = 8.7, 7.2, 1.7 Hz, 1H), 7.50–7.30 (m, 3H), 6.99 (d, *J* = 1.0 Hz, 1H), 6.96 (d, *J* = 2.0 Hz, 1H), 6.88 (d, *J* = 8.2 Hz, 1H), 6.84–6.76 (m, 3H), 6.74 (d, *J* = 1.8 Hz, 1H), 3.92 (s, 3H), 3.89 (s, 3H), 3.85 (s, 3H), 3.82 (s, 3H). ^13^C NMR (75 MHz, CDCl_3_) δ 176.6, 156.2, 152.8, 149.1, 148.8, 142.9, 133.6, 126.4, 125.3, 125.1, 124.5, 124.5, 121.1, 118.1, 112.4, 112.3, 111.2, 56.0, 56.0. HRMS (ESI^+^) [M + H]^+^ calculated for C_25_H_20_O_6_, 445.1646; found, 445.1649.

**3-[2,2-Bis(4-chlorophenyl)vinyl]-6-chloro-4*H*-chromen-4-one (3h)**: ^1^H NMR (300 MHz, CDCl_3_) δ 8.22 (dd, *J* = 2.6, 0.4 Hz, 1H), 7.59 (dd, *J* = 8.9, 2.6 Hz, 1H), 7.42–7.27 (m, 5H), 7.23 (s, 1H), 7.21–7.11 (m, 4H), 6.99 (d, *J* = 1.1 Hz, 1H).^13^C NMR (75 MHz, CDCl_3_) δ 175.9, 155.0, 154.8, 142.5, 140.1, 137.8, 134.2, 134.0, 133.9, 131.3, 131.3, 129.5, 128.9, 128.5, 125.5, 124.6, 121.7, 119.9, 117.8. HRMS (ESI^+^) [M + H]^+^ calculated for C_23_H_14_Cl_3_O_6_, 427.0054; found, 427.0042.

**7-(Benzyloxy)-3-[2,2-bis(4-chlorophenyl)vinyl]-4*H*-chromen-4-one (3i):**^1^H NMR (300 MHz, CDCl_3_) δ 8.17 (d, *J* = 8.9 Hz, 1H), 7.47–7.31 (m, 8H), 7.31-7.26 (m, 2H), 7.24–7.13 (m, 4H), 7.05 (dd, *J* = 8.9, 2.4 Hz, 1H), 7.02 (d, *J* = 1.2 Hz, 1H), 6.80 (d, *J* = 2.3 Hz, 1H), 5.13 (s, 2H). ^13^C NMR (75 MHz, CDCl_3_) δ 177.4, 155.8, 154.8, 149.5, 149.2, 148.7, 148.6, 143.7, 135.4, 133.6, 132.6, 126.2, 125.2, 123.8, 122.4, 122.2, 120.8, 118.2, 115.3, 112.8, 111.6, 110.7, 110.6, 56.0. HRMS (ESI^+^) [M + H]^+^ calculated for C_30_H_21_Cl_2_O_3_, 499.0862; found, 499.0860.

**3-[2,2-Bis(3,4-dimethoxyphenyl)vinyl]-6-methyl-4*H*-chromen-4-one (3j):**^1^H NMR (300 MHz, CDCl_3_) δ 8.04 (dd, *J* = 2.2, 1.0 Hz, 1H), 7.43 (dd, *J* = 8.6, 2.2 Hz, 1H), 7.36 (d, *J* = 1.0 Hz, 1H), 7.23 (s, 1H), 7.19 (t, *J* = 2.4 Hz, 1H), 7.04–6.97 (m, 2H), 6.92-6.85 (m, 2H), 6.83–6.79 (m, 2H), 3.92 (s, 3H), 3.89 (s, 3H), 3.84 (s, 3H), 3.74 (s, 3H), 2.45 (s, 3H). ^13^C NMR (75 MHz, CDCl_3_) δ 177.4, 154.7, 154.0, 149.3, 149.0, 148.6, 148.4, 143.4, 135.3, 135.1, 130.7, 130.4, 125.3, 125.1, 123.3, 122.4, 120.7, 117.8, 115.4, 112.8, 111.4, 110.6, 110.5, 56.0, 55.9, 55.8, 21.0. HRMS (ESI^+^) [M + H]^+^ calculated for C_26_H_23_O_4_, 399.1591; found, 399.1598.

## 4. Conclusions

In summary, we have developed an environmentally benign, economically friendly, and sustainable Suzuki–Miyaura reaction of bromochromones and boronic acids employing Pd(II) immobilized in a silica-supported ionic liquid in water using ohmic heating.

This method offers significant improvements over existing procedures, as the absence of undesired side reactions, the short reaction times, the mild conditions required, the wide range of functionalities tolerated, the good yields, the environment-friendly reaction conditions, and the low catalyst loading. In addition, the supported catalyst can be recovered and maintains a good activity for at least three cycles.

Thus, the combination of ohmic heating with supported ionic liquid phase catalysis (SILPC) in water not only is of considerable interest for cross-coupling reactions but also provides a convenient alternative to the existing methodologies for the synthesis of flavonoid derivatives, including isoflavones, 3-stryrylchromones, and the elusive 3-diarylvinylchromones.

## Figures and Tables

**Table 1:**
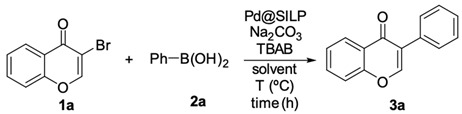
Suzuki–Miyaura reaction of 3-bromochromone **1a** and phenylboronic acid **2a**.

Entry	Solvent	mol% Pd	TBAB (mol%)	T (°C)	Time (h)	Yield (%) ^a^
1	bmim[Br]	1	-	100 ^b^	12	34
2	bmim[PF_6_]	1	-	100 ^b^	12	65
3	DMF	1	-	100 ^b^	12	69
4	DMF/H_2_O 5:2	1	-	100 ^b^	12	72
5	H_2_O	1	-	100 ^b^	12	9
6	H_2_O	1	0.1	100 ^b^	12	89
7	H_2_O	1	0.1	100 ^c^	1	93
8	H_2_O	0.1	0.1	100 ^c^	1	92
9	H_2_O	0.05	0.1	100 ^c^	1	90

*^a^* isolated yield; *^b^* oil bath; *^c^* ohmic heating.

**Table 2:**
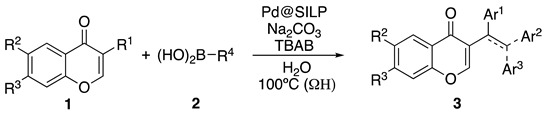
Suzuki–Miyaura reactions of bromoflavones **1** with arylboronic acids **2**.

Entry	R^1^	R^2^	R^3^	1	R^4^	2	Product	3	Yield (%)^a^
1	Br	H	H	**1a**	Ph	2a	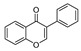	3**a**	93
2	Br	H		**1a**	3,4-OMeC_6_H_3_	**2b**	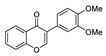	3**b**	89
3	Br	H	H	**1a**	4-ClC_6_H_4_	**2c**	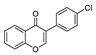	3c	91
4	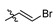	H	H	1b	Ph	**2a**	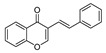	3d	90
5	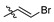	H	H	**1b**	3,4-OMeC_6_H_3_	2b	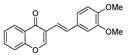	3e	85
6	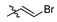	H	OBn	**1c**	3,4-OMeC_6_H_3_	**2b**	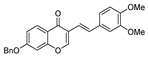	3f	81
7	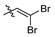	H	H	**1d**	3,4-OMeC_6_H_3_	**2b**	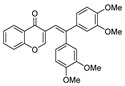	3**g**	82
8	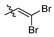	Cl	H	**1e**	4-ClC_6_H_4_	**2c**	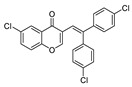	3h	89
9	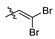	H	OBn	**1f**	4-ClC_6_H_4_	**2c**	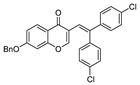	3**i**	85
10	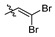	Me	H	**1g**	3,4-OMeC_6_H_3_	**2b**	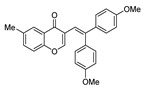	3**j**	80

*^a^* Isolated yield.

## Supplementary Files

Supplementary File 1
