# Supplementary material for: Ionic Liquids and Ohmic Heating in Combination for Pd-Catalyzed Cross-Coupling Reactions: Sustainable Synthesis of Flavonoids
Source: Molecules. 2020 Mar 29;25(7):1564. doi: 10.3390/molecules25071564 (PMC7180935; doi:10.3390/molecules25071564)

molecules

Supplementary Material

Ionic Liquids and Ohmic Heating in Combination for Pd-catalyzed Cross-coupling Reactions: Sustainable Synthesis of Flavonoids

Vera L. M. Silva [1], Raquel G. Soengas [2] and Artur M. S. Silva [1,*]

1 LAQV-REQUIMTE, Department of Chemistry, University of Aveiro, 3810-193 Aveiro, Portugal; verasilva@ua.pt
2 Department of Organic and Inorganic Chemistry, University of Oviedo, c/Julián Clavería 6, 33006 Oviedo, Spain; rsoengas@uniovi.es
* Correspondence: artur.silva@ua.pt

Academic Editor: Vito Capriati
Received: 24 February 2020; Accepted: 26 March 2020; Published: date.

Figure S1. 3-Phenyl-4*H*-chromen-4-one (3a)

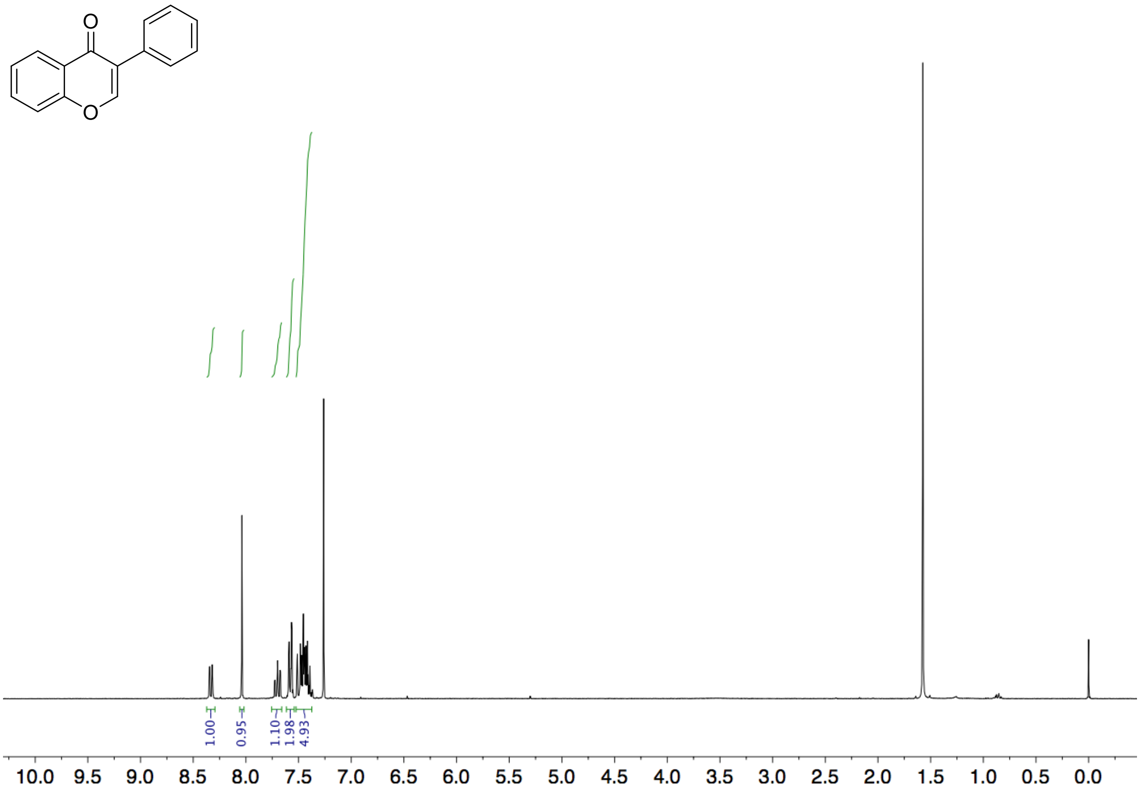

Figure S2. 3-(3,4-Dimethoxyphenyl)-4*H*-chromen-4-one (3b)

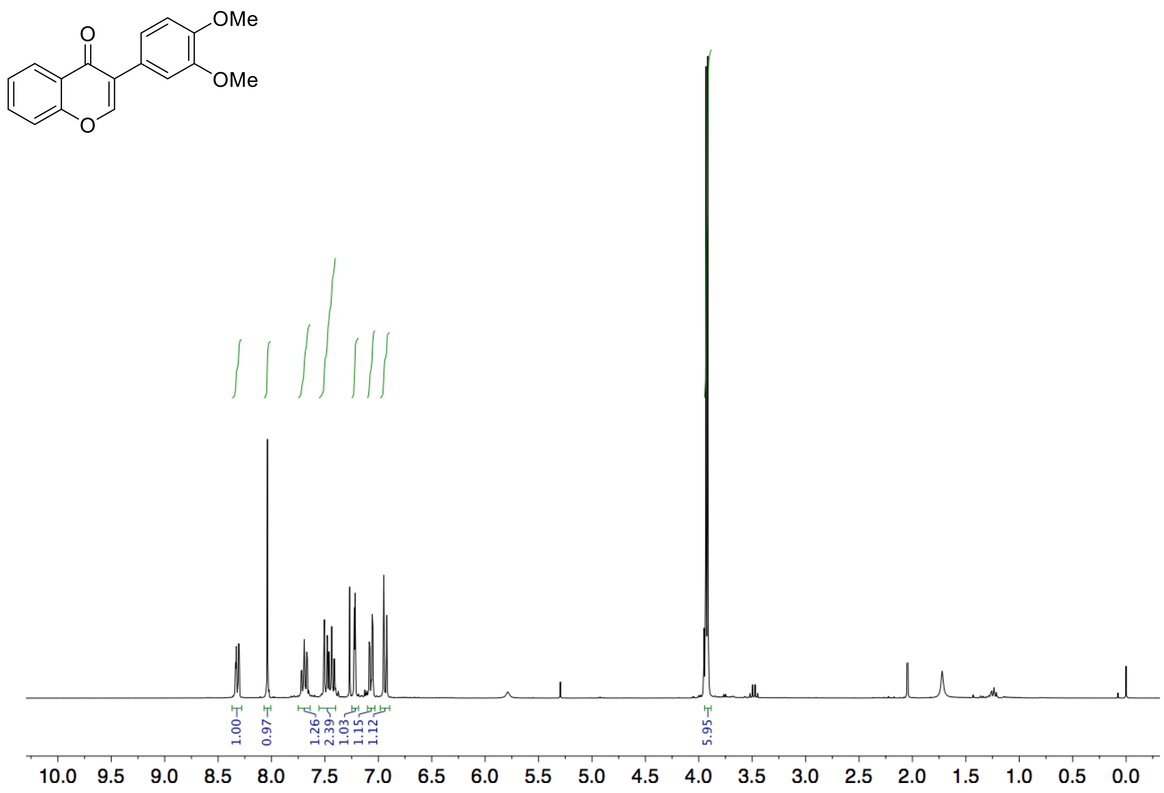

Figure S3. **3-(4-Chlorophenyl)-4*H*-chromen-4-one (3c)**

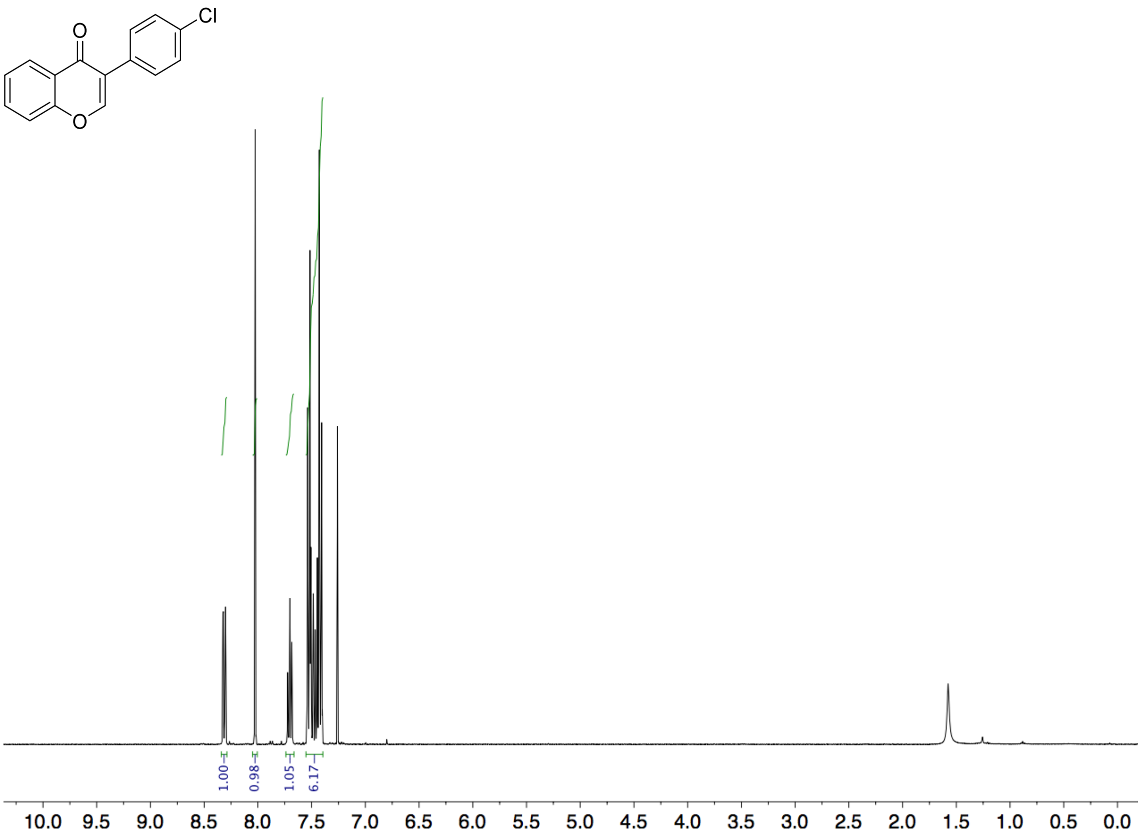

Figure S4. **3-Styryl-4*H*-chromen-4-one (3d)**

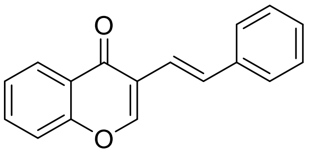

Figure S5. **3-(3,4-Dimethoxystyryl)-4*H*-chromen-4-one (3e)**

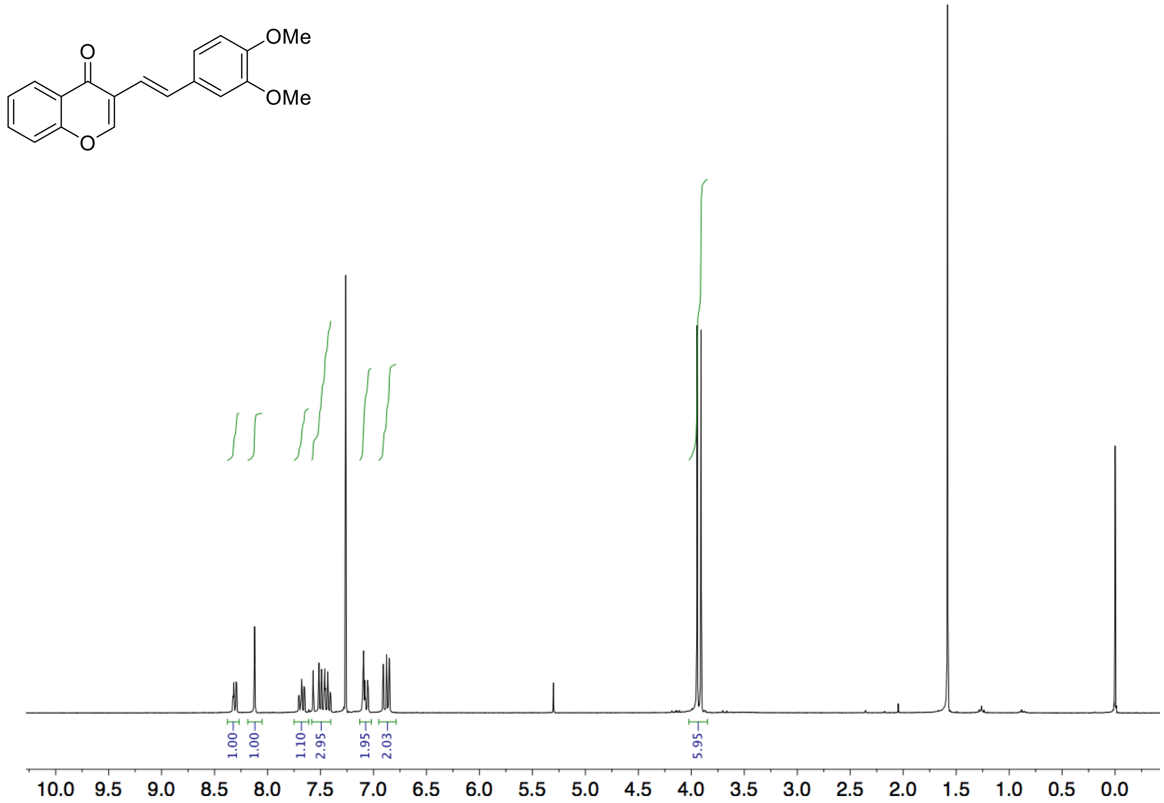

Figure S6. 7-(Benzyloxy)-3-(3,4-dimethoxystyryl)-4*H*-chromen-4-one (3f)

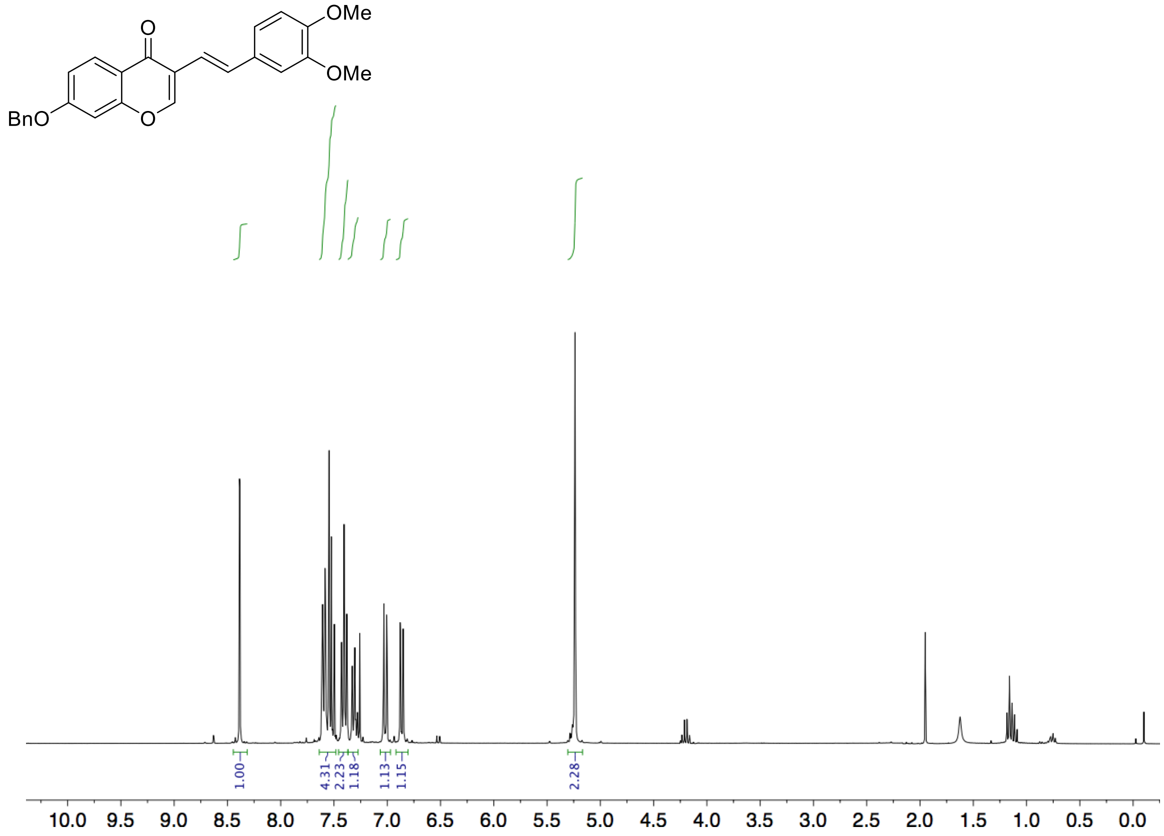

Figure S7. 3-(2,2-Bis(3,4-dimethoxyphenyl)vinyl)-4*H*-chromen-4-one (3g)

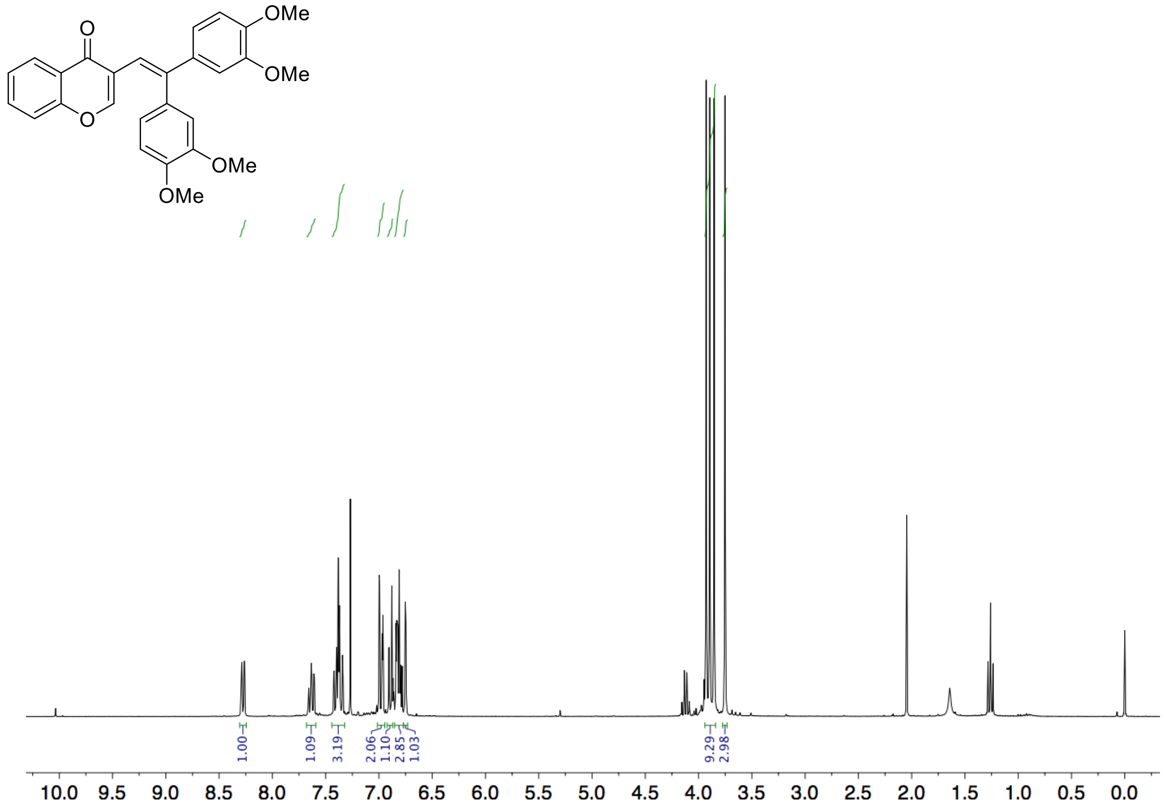

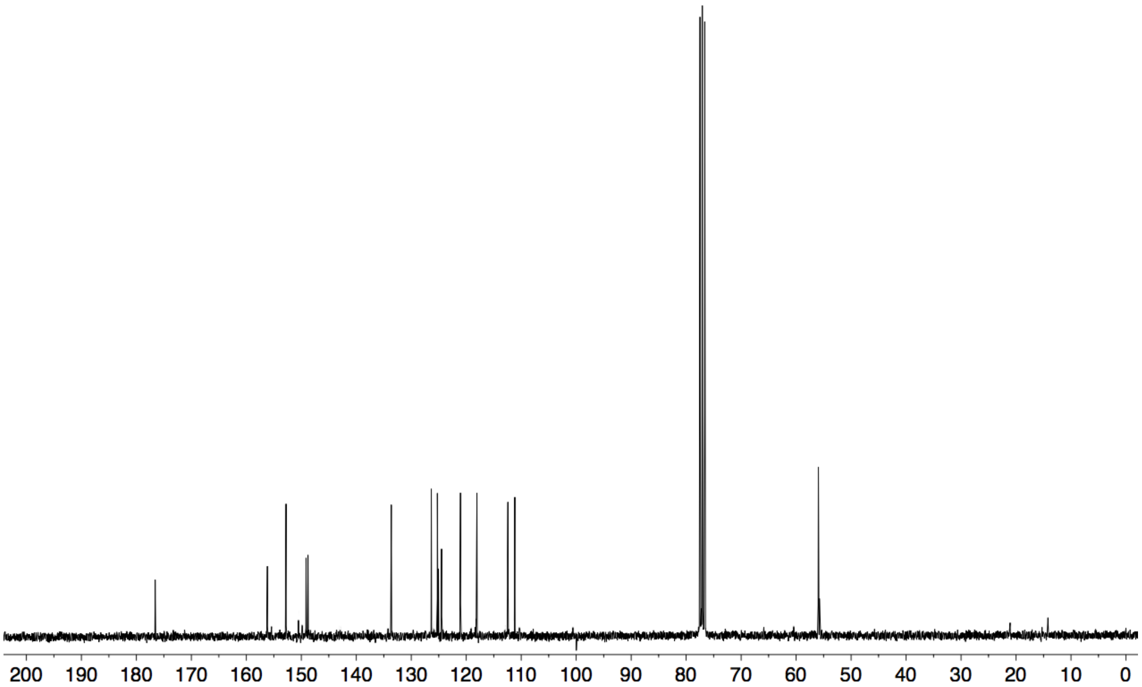

Figure S8. 3-(2,2-Bis(4-chlorophenyl)vinyl)-6-chloro-4*H*-chromen-4-one (3h)

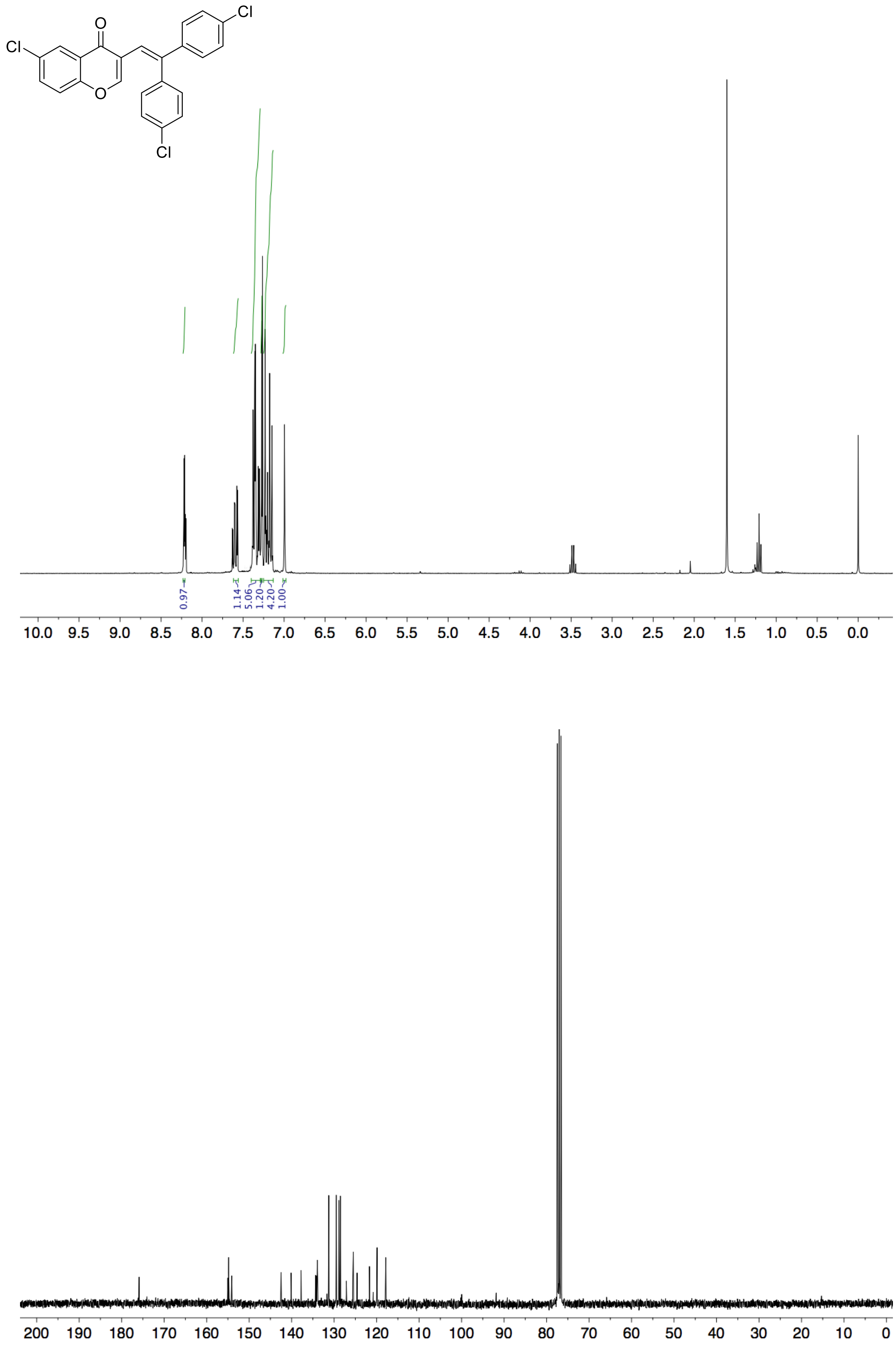

Figure S9. 7-(Benzyloxy)-3-(2,2-bis(4-chlorophenyl)vinyl)-4*H*-chromen-4-one (3i)

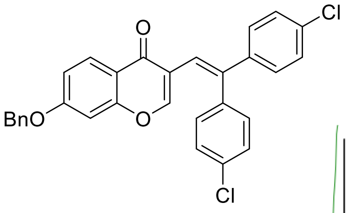

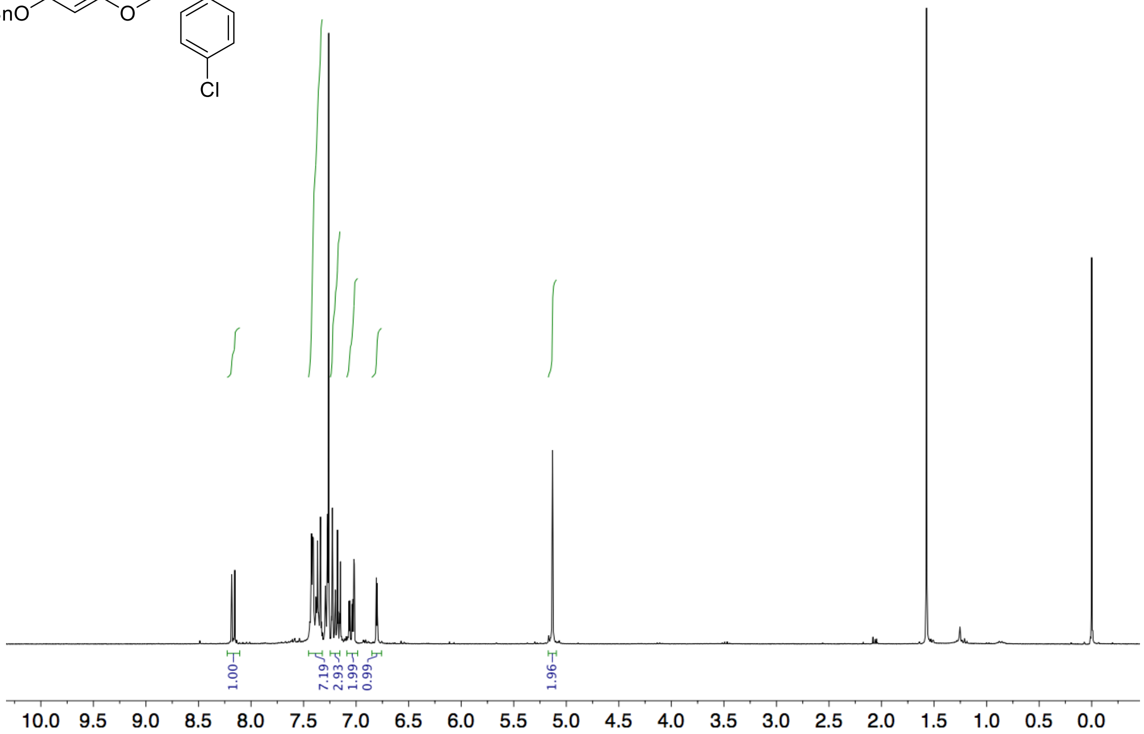

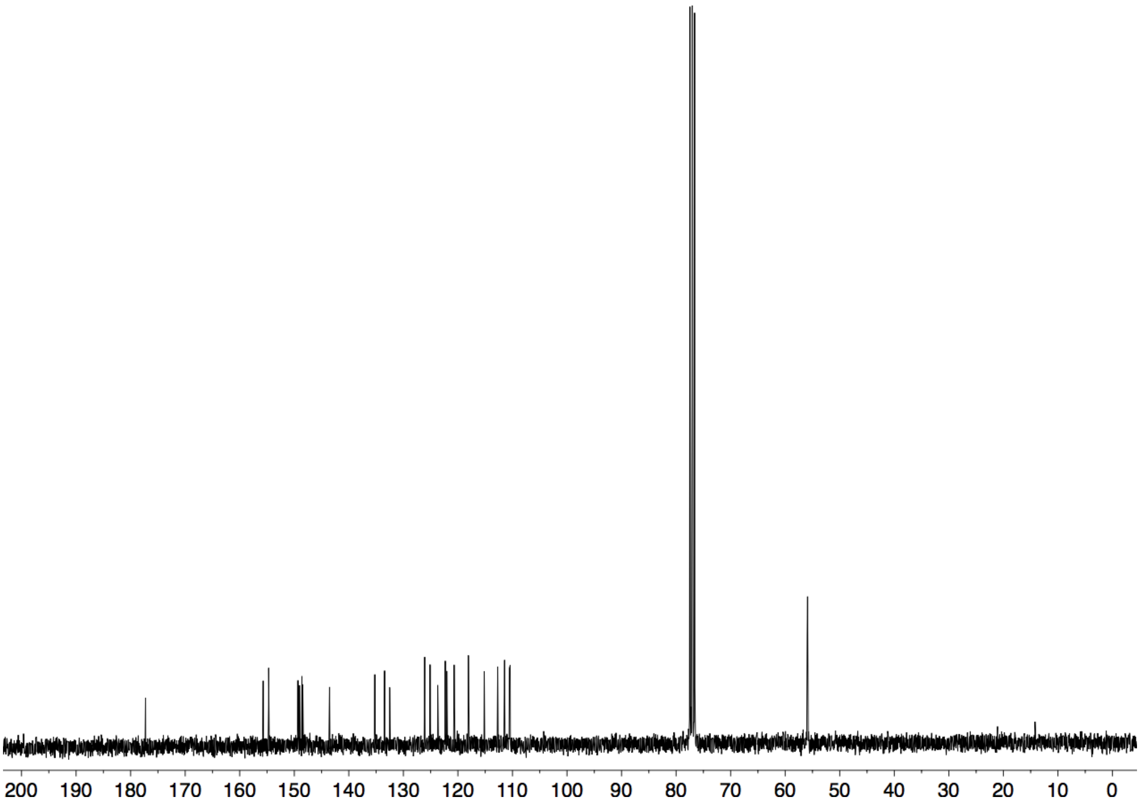

Figure S10. 3-(2,2-Bis(3,4-dimethoxyphenyl)vinyl)-6-methyl-4*H*-chromen-4-one (3j)

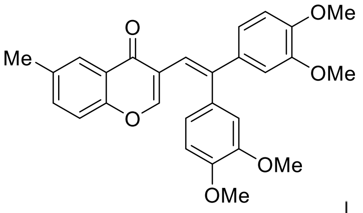

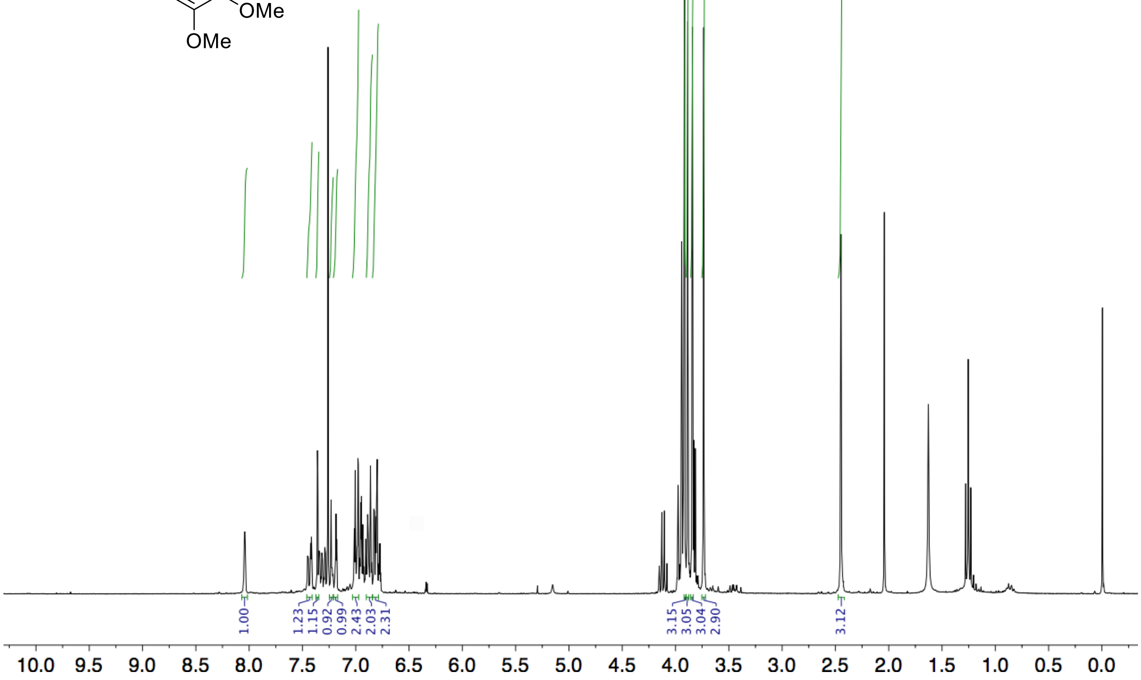

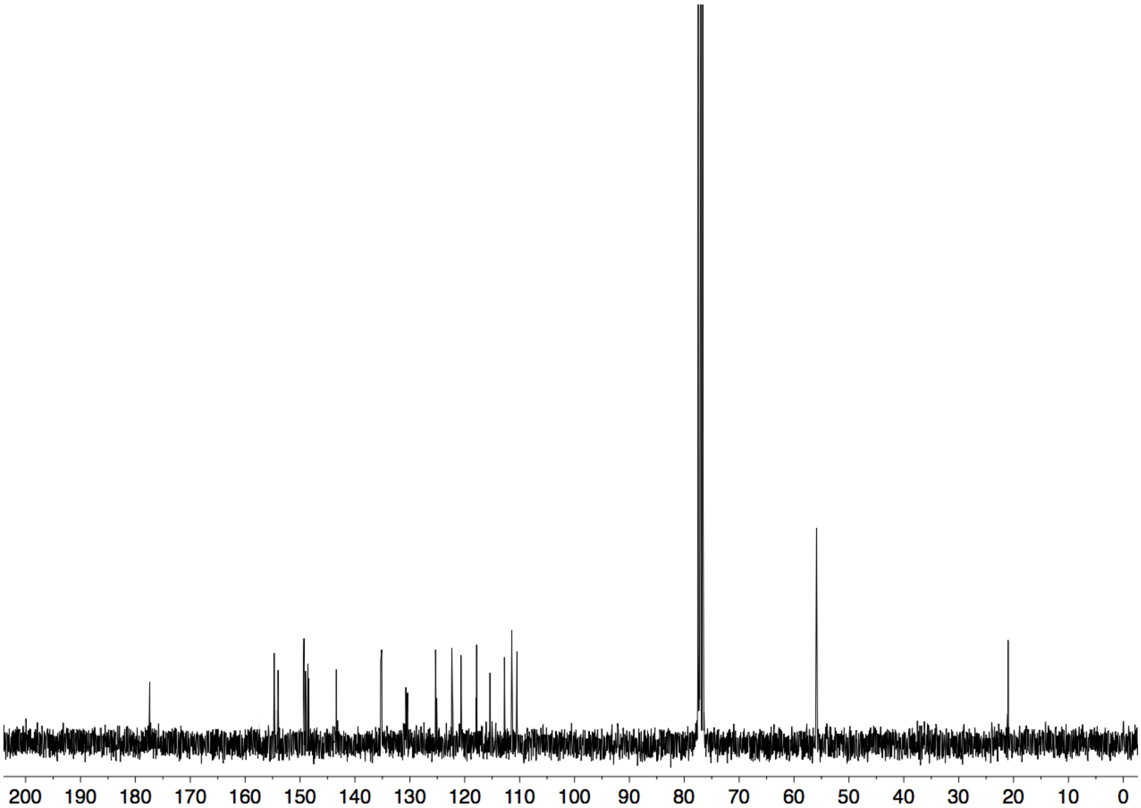

Supplement: Supplementary File 1 [file molecules-25-01564-s001.pdf]
